# Association between dietary selenium intake and endometriosis risk: a cross-sectional analysis

**DOI:** 10.3389/fendo.2025.1486790

**Published:** 2025-06-30

**Authors:** Jia-Jie Guo, Rui-Xuan Li, Wen-Li Shang, Ya-Fang Zheng, Guo-Yi Zhu, Zhou-Chang Shu, Gui-Chao Liu, Hong-Biao Ou, Jia-Ying Li, Xu-Guang Guo, Li-Hong Lin

**Affiliations:** ^1^ Department of Obstetrics and Gynecology, Center for Reproductive Medicine; The Third Affiliated Hospital, Guangzhou Medical University, Guangzhou, China; ^2^ Guangdong Provincial Key Laboratory of Major Obstetric Diseases; The Third Affiliated Hospital, Guangzhou Medical University, Guangzhou, China; ^3^ Guangdong Provincial Clinical Research Center for Obstetrics and Gynecology; The Third Affiliated Hospital, Guangzhou Medical University, Guangzhou, China; ^4^ Guangdong-Hong Kong-Macao Greater Bay Area Higher Education Joint Laboratory of Maternal-Fetal Medicine, The Third Affiliated Hospital, Guangzhou Medical University, Guangzhou, China; ^5^ The Second School of Clinical Medicine, Guangzhou Medical University, Guangzhou, China; ^6^ School of Health Management, Guangzhou Medical University, Guangzhou, China; ^7^ Department of Clinical Medicine, The Third School of Clinical Medicine, Guangzhou Medical University, Guangzhou, China; ^8^ School of Basic Medical Sciences, Guangzhou Medical University, Guangzhou, China; ^9^ Institute of Gerontology, Guangzhou Geriatric Hospital, Guangzhou Medical University, Guangzhou, China; ^10^ State Key Laboratory of Respiratory Disease, Guangzhou Geriatric Hospital, Guangzhou Medical University, Guangzhou, China; ^11^ Collaborative Innovation Center for Civil Affairs of Guangzhou, Guangzhou, China; ^12^ Department of Clinical Laboratory Medicine, Guangzhou Geriatric Hospital, Guangzhou Medical University, Guangzhou, China

**Keywords:** selenium, dietary selenium intake, endometriosis, diet, cross-sectional study, NHANES

## Abstract

**Background:**

Endometriosis (EMs) is a common chronic inflammatory disorder with estrogen dependency, and its causes and progression are not fully understood. With limited treatment options available, the dietary impact on EMs incidence has gained research interest. This study explores the link between dietary selenium intake and EMs risk, noting selenium’s key antioxidant role in reducing oxidative stress and inflammation, and its potential to modulate immune responses, offering protective effects.

**Methods:**

The study included 39,352 participants from National Health and Nutrition Examination Survey (NHANES) data (1999-2006). We excluded individuals with missing data on dietary selenium intake or EMs status, pregnant women, and individuals with missing basic covariate data or suspected erroneous dietary selenium intake values. After these exclusions, a final cohort of 3,876 participants was included for detailed analysis. This cohort was stratified into two groups: 3566 individuals without a diagnosis of endometriosis and 310 individuals diagnosed with EMs. The relationship between EMs and dietary selenium intake was examined using a suite of statistical methodologies, including multivariate logistic regression to control for confounding variables, smooth curve fitting, threshold effect analysis and subgroup analysis.

**Results:**

After adjusting for multiple covariates, the multivariate logistic regression model indicated a negative correlation between dietary selenium intake and the risk of developing EMs. In the highest dietary selenium intake group, the adjusted model II revealed a reduction in the risk of EMs by approximately 34.1% (OR = 0.659, 95% CI: 0.449, 0.967). The subgroup analysis revealed a negative relationship between quartiles of selenium intake and the risk of endometriosis in participants aged fifty years and older, in non-Hispanic white participants, in participants with PIR >=1.3 and <3.5, in participants with a high school education level or under, in participants who get married or live with a partner, in participants who have never drunk, and in participants who smoke currently.

**Conclusions:**

Our findings suggest a negative correlation between dietary selenium intake and endometriosis risk. However, potential confounding factors may influence this association. Given the limitations of this cross-sectional study, such as reliance on self-reported data, further prospective research is required to confirm causality and explore underlying mechanisms.

## 1 Introduction

Endometriosis (EMs) is a common inflammatory disease characterized by the presence of tissue resembling endometrium outside the uterus, primarily affecting pelvic organs and tissues. Approximately 176 million women worldwide suffer from this condition, with 5%-10% of fertile women experiencing pelvic pain and infertility (1). EMs increases the medical burden and mental stress on the reproductive-age population, making the search for effective prevention and treatment measures particularly important.

The study found that the imbalance between the oxidative system and the antioxidant system and the increase of oxidation markers in patients with EMs showed that antioxidants had preventive and therapeutic effects on EMs, indicating that EMs were related to oxidative stress (2). In addition, the increase of reactive oxygen species leads to damage to DNA, proteins, and lipids, which in turn promotes inflammation and cell proliferation, and previous clinical studies have confirmed the close relationship between inflammatory cytokines and EMs, suggesting that inflammatory factors can be used as predictors of EMs (3). Studies have shown that the intake of antioxidant foods can reduce oxidative damage and maintain the normal function of cells; Consuming a diet rich in anti-inflammatory ingredients can modulate the body’s inflammatory response (4).

Selenium (Se), as a trace element, has long been recognized as an important food-based antioxidant, showing high activity in antioxidant and anti-inflammatory activities (5). Due to the large atomic radius of Se, its outer electrons are easily lost, endowing selenide compounds with a -2 valence excellent antioxidant effects. Selenium can eliminate reactive oxygen species (ROS) through single-electron or double-electron reduction mechanisms and combat oxidative stress by activating the Keap1-Nrf2-ARE signaling pathway (6). Studies have shown that selenium is an important component of the antioxidant enzyme that is used by the glutathione peroxidase (GPX) system to eliminate different ROS and is one of the major sulfhydryl-dependent antioxidant systems that help the body fight oxidative stress (7). It has been found that GPX, as a selenium-containing enzyme, is widely distributed in human epithelial cells, participates in the elimination of hydroxyl radicals, effectively reduces oxidative stress, and has a certain protective effect on EMs (8).

With the continuous development of society, dietary selenium has become the main source of selenium in the human body. Multiple studies have confirmed that selenium-rich products, including selenium-enriched brown rice (9), selenium-enriched spirulina (10), selenium-enriched oolong tea (11), etc., exert anti-inflammatory effects through the NF-κ B/MAPK signaling pathway. Wang et al. have found that selenoproteins extracted from dietary selenium can effectively inhibit the production of IL-6, TNF-α, etc. (12). GPX plays an important role in the human body as one of the selenoproteins (13). Selenium deficiency activates the mitogen-activated protein kinase (MAPK) pathway, resulting in an increase in pro-inflammatory factors and a decrease in anti-inflammatory factors (12). Studies using animal models have found that selenium compounds can inhibit the onset and progression of inflammation (14), so it can be inferred that it may have some protective effect on EMs. In addition, due to its excellent ROS clearance ability, selenium has potential applications in the treatment of related diseases.

Through our investigation and research, there is currently no clear study indicating that dietary selenium directly affects the occurrence or development of EMs, but relevant studies have shown that dietary selenium plays an important role in antioxidant and anti-inflammatory. Therefore, with the help of public data from the National Health and Nutrition Examination Survey (NHANES), we selected a dataset from 1999-2006 as the basis for our analysis, which aimed to investigate the link between dietary cholesterol intake and the risk of developing endometriosis in adult women in general. The goal is to provide evidence that informs the development of effective treatment strategies. At the time of our study, the NHANES data up to 2006 was the most recent dataset that included information on endometriosis. Although NHANES has published updated data for 2021-2023, these updates did not include endometriosis-related information. Therefore, the 1999-2006 dataset remains the most appropriate and comprehensive for our analysis.

## 2 Methods

### 2.1 Data source and ethics

The NHANES, a program created especially to collect data on health, nutrition, and laboratory results from a cross-sectional sample of the non-institutionalized U.S. population, provided all of the materials and data used in this study. Trained professionals used questionnaires, health interviews, and laboratory testing to gather participant demographic, health status, and laboratory data.

### 2.2 Study population

**Figure 1:**
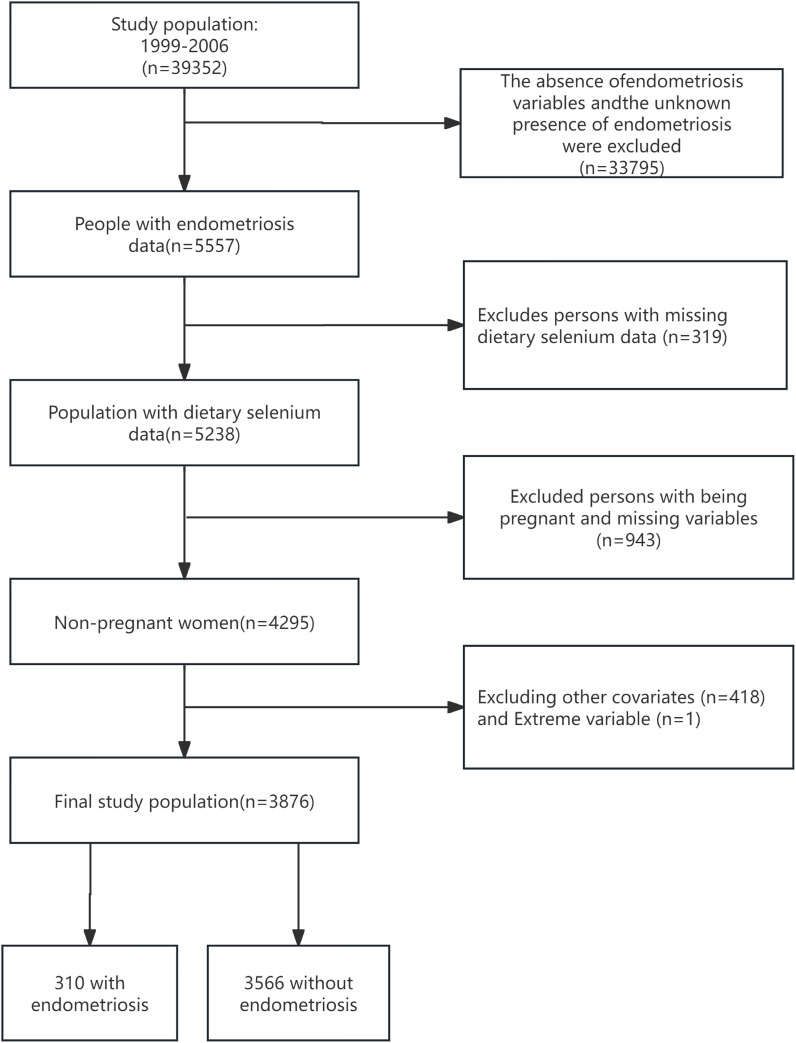
Summary of the inclusion and exclusion criteria of study subjects.

Focusing on EMs from 1999 to 2006, this study utilized NHANES data from four cycles: 1999-2000, 2001-2002, 2003-2004, and 2005-2006, including a total of 39,352 participants. Initially, individuals with missing EM variables or unknown EMs status (n=33,795), and those lacking dietary selenium intake data (n=319), were excluded to ensure data integrity. Considering the significant impact of EMs on fertility, pregnant women were excluded from data collection to improve study accuracy (15). After systematically excluding pregnant women and those with unclear pregnancy status (n=943), as well as individuals lacking basic covariate data (Age; Race; BMI; Poverty Income Ratio (PIR); Education level; Marital status; Alcohol consumption; Smoking status; Hypertension; Hyperlipidemia; Vitamin B12; physical activity (PA); Ever taken birth control; Regular period), and those with suspected erroneous dietary selenium intake values (data=0), a final cohort of 3876 participants was included for detailed analysis, as depicted in **Figure 1**.

### 2.3 Exposure variable

In this study, the exposure variable was dietary selenium. NHANES collected non-consecutive two-day food intake data, with the first interviews conducted via questionnaires and the second via telephone survey. During these surveys, participants provided dietary details of their intake within 24 hours, and the investigators estimated the average food intake over two days, utilizing the United States Department of Agriculture’s food and nutrient database for dietary studies to estimate nutrient content (16). During the period from 1999 to 2002, only the first day’s dietary recall data were publicly accessible, and thus, only the amount of food intake reported on the first day was utilized in this study. To ensure the quality and accuracy of the interviews, all dietary interviewers were required to complete a rigorous one-week training program and conduct supervised practical interviews before being permitted to conduct interviews independently. After a normality test, which confirmed the non-normal distribution of the data, we found notable skewness in the dietary selenium exposure variable. By utilizing a Box-Cox transformation on the dietary selenium variable data, we aimed to better approximate a normal distribution, ultimately improving the accuracy of our statistical results through the correction of identified skewness. The Box-Cox transformation is a widely used statistical method that transforms non-normal dependent variables into a normal distribution, which is essential for many statistical techniques, particularly for nutrient variables (17). For detailed information on the Box-Cox transformation, please refer to **Supplementary Material S1**.

### 2.4 Outcome variable

Data on EMs were obtained by NHANES through a questionnaire asking participants whether they had been informed through examination that they had EMs, specifically inquiring whether they had been formally diagnosed with EMs by a doctor or other healthcare professional. If the questionnaire response was “yes,” the participant was considered to have EMs (18). Self-reported endometriosis has been demonstrated to possess a high degree of accuracy, with over 95% of self-reported cases being confirmed as laparoscopy-verified endometriosis (19).

### 2.5 Covariates

For the sake of achieving higher data precision and thorough analysis, this study took into account various factors such as age, race, BMI, PIR, education level, marital status, alcohol consumption, smoking status, hyperlipidemia, hypertension, theobromine intake, vitamin B12 intake, carbohydrate intake, PA, ever taken birth control and regular period. Age was split into less than 40, 40 to less than 50, and greater than 50. Participants were divided into groups based on race: Mexican American, other Hispanic, non-Hispanic white, non-Hispanic black, and Other Race (Including Multi-Racial) (20). BMI measurements were taken at the Mobile Examination Center (MEC) and converted into less than 25, 25 to less than 30, or greater than 30 (21). To assess household income, the study chose to use PIR, a measure calculated based on family size thresholds. Poverty income ratio was split into less than 1.3, 1.3 to less than 3.5, and greater than 3.5 (22). We classified education level as under high school, high school, and more than high school (23). Marital status was sorted into Married/Living with a partner, Never married, and Divorced/Separated/Widowed. Alcohol consumption was sorted into former drinker, never drinker, mild drinker, moderate drinker, and heavy drinker. Smoking status was classified as never smoking, former smoking, and now smoking (24). Participants underwent dietary recall interviews before the MEC interview to obtain their 24-hour nutrition data, including theobromine intake, vitamin B12 intake, carbohydrate intake, etc. The assessment of PA was determined by the MET (Metabolic Equivalent of Task) values, which were derived from the type, frequency, and duration of activities performed per week. The calculation was performed using the following formula: PA (MET-min/wk) = MET × weekly frequency × duration of each physical activity (25). A value of PA=0 indicates participants who do not engage in any physical activity; any other value signifies that participants have either consistent or intermittent physical activity. Participants were categorized based on whether they met the American physical activity guidelines, which recommend that moderate-intensity physical activity should be performed for 150 minutes per week (equivalent to 600 MET-min/wk) or, for vigorous-intensity activity, 75 minutes per week for adults (26). “Ever taken birth control” was included as a covariate in the analysis. Additionally, “menstrual cycle regularity in the past year” was also considered as a covariate. All covariate data in this study were derived from statistical analysis on the NHANES website. The necessary steps for acquiring selenium intake and other related factors are accessible at http://cdc.gov/nchs/nhanes.

### 2.6 Statistical analysis

The statistical methods employed include descriptive analysis of the study population, stratified analysis, multiple logistic regression, smoothing curve fittings and threshold effect analysis. “Mean ± Standard Deviation (SD)” or “Median(Q1-Q3)” is used to represent continuous variables in this study, whereas categorical variables are displayed with numbers and percentages (%). P values were calculated by one-way ANOVA (normal distribution) and the Kruskal–Wallis H (skewed distribution) test, and the median (Q1-Q3) was used only when the mean value was less than double the standard difference. The chi‐square test is used for categorical variables.

To further investigate the impact of varying selenium levels and other potential confounding factors on the relationship between EMs and selenium Box-Cox, selenium Box-Cox group, and selenium Box-Cox group trend, selenium intake was categorized into four groups: Q1 (-1.109 - 9.830), Q2 (9.831 - 11.375), Q3 (11.377 - 12.819) and Q4 (12.820 - 29.253). Multivariate logistic regression analysis was then conducted for each group. Age, race, PIR, education level, and marital status were controlled for in the Adjusted I model, while the Adjusted II model accounted for BMI, alcohol consumption, smoking status, hypertension, hyperlipidemia, theobromine intake, vitamin B12 intake, PA, ever taken birth control and regular period.

Additionally, this research, utilizing the unadjusted model and adjusted II model, utilized smooth curve fittings to illustrate the connection between dietary selenium intake and the risk of EMs and uncover any nonlinear associations. Threshold effect analysis was conducted to explore the implicit nonlinear relationship in the smooth curve fitting.

To investigate whether there were interactions and stability between subgroups, we performed subgroup analyses for age, race, PIR, BMI, education level, marital status, alcohol consumption, smoking status, hyperlipidemia and hypertension. If the P for interaction across different stratifications is >0.05, it suggests the results are reliable across different subgroups; otherwise, it may indicate the presence of special populations (27).

Stratified analyses of the curve analysis were also conducted based on age, PIR, education level, marital status, alcohol consumption and smoking Status to visually describe the relationship between dietary selenium intake and the risk of EMs across different populations. Threshold effect analysis was also conducted to explore the implicit nonlinear relationship in the smooth curve fitting.

Finally, to ensure the robustness of the results, sensitivity analysis was performed in this study. After excluding extreme outliers with dietary selenium intake greater than 400, the multivariate logistic regression analysis was repeated.

The statistical software EmpowerStats (www.empowerstats.com) was utilized in conjunction with the R statistical programming language (X64 version 4.2.0; R Foundation for Statistical Computing) to process and analyze the entire dataset. P < 0.05 was used to indicate statistical significance.

## 3 Results

### 3.1 Participant characteristics

The descriptive characteristics of the participants are presented in **Table 1**. The analysis of **Table 1** highlighted a significant difference in the mean dietary selenium intake between the non-EMs group and the EMs group, with the former exhibiting a higher intake (p < 0.05). Differences in age, race, PIR, BMI, educational level, marital status, alcohol consumption, smoking status, hypertension, ever taken birth control, and regular menstrual cycle between the EMs group and the non-EMs group were statistically significant (p < 0.05). Compared to the non-EMs group, the EMs group had a higher overall age, a higher proportion of Hispanic Whites, a higher PIR, a lower BMI, a higher proportion of married/living with partner and divorced/separated/widowed individuals, a higher educational level, a higher proportion of individuals who had ever taken birth control, and a higher proportion of individuals with irregular menstrual cycles in the past year. No significant differences or no differences were observed between the two groups for the remaining variables.

**Table 1:** Baseline population characteristics.

Variables	Non-Endometriosis	Endometriosis	P-value
Participants	3566	310	
Age (years)	37.225 ± 10.090	41.229 ± 8.323	**<0.001**
Theobromine (mg)	8.000 (0.000-46.000)	11.395 (0.000-51.750)	0.063
Vitamin_B12 (μg)	3.345 (1.991-5.310)	3.228 (2.120-4.862)	0.397
Carbohydrate (g)	225.208 (168.319-294.036)	212.410 (164.721-283.973)	0.074
Selenium (mcg)	87.625(63.912-113.900)	80.900(57.368-107.188)	**0.007**
Selenium Box-Cox	11.393 + 2.638	11.030 + 2.515	**0.007**
Age, n(%)			**<0.001**
<40	1937 (54.319%)	121 (39.032%)	
>=40, <50	1127 (31.604%)	129 (41.613%)	
>=50	502 (14.077%)	60 (19.355%)	
Race, n (%)			**<0.001**
Mexican American	815 (22.855%)	27 (8.710%)	
Other Hispanic	789 (22.126%)	56 (18.065%)	
Non-Hispanic White	1643 (46.074%)	212 (68.387%)	
Non-Hispanic Black	177 (4.964%)	6 (1.935%)	
Other Race (Including Multi-Racial)	142 (3.982%)	9 (2.903%)	
PIR	2.645 ± 1.644	3.133 ± 1.627	**<0.001**
PIR, n (%)			**<0.001**
<1.35	1021 (28.632%)	61 (19.677%)	
>=1.35, <3.5	1310 (36.736%)	103 (33.226%)	
>=3.5	1235 (34.633%)	146 (47.097%)	
BMI (kg/m²)	28.895 ± 7.542	28.663 ± 7.139	0.757
BMI (kg/m²), n (%)			0.687
<25	1282 (35.951%)	112 (36.129%)	
>=25, <30	943 (26.444%)	88 (28.387%)	
>=30	1341 (37.605%)	110 (35.484%)	
Education Level, n (%)			**<0.001**
Under High School	827 (23.191%)	39 (12.581%)	
High School	781 (21.901%)	86 (27.742%)	
More than High School	1958 (54.907%)	185 (59.677%)	
Marital Status, n (%)			**<0.001**
Married/Living with partner	2181 (61.161%)	204 (65.806%)	
Never Married	776 (21.761%)	37 (11.935%)	
Divorced/Seperated/Widowed	609 (17.078%)	69 (22.258%)	
Alcohol Consumption, n (%)			**0.009**
Never	605 (16.966%)	34 (10.968%)	
Former	522 (14.638%)	57 (18.387%)	
Mild	903 (25.322%)	96 (30.968%)	
Moderate	766 (21.481%)	64 (20.645%)	
Heavy	770 (21.593%)	59 (19.032%)	
Smoking Status, n (%)			**0.007**
Never	2198 (61.638%)	163 (52.581%)	
Former	529 (14.835%)	58 (18.710%)	
Now	839 (23.528%)	89 (28.710%)	
Hypertension, n (%)			**<0.001**
No	2742 (76.893%)	203 (65.484%)	
Yes	824 (23.107%)	107 (34.516%)	
Hyperlipidemia, n (%)			0.573
No	1288 (36.119%)	107 (34.516%)	
Yes	2278 (63.881%)	203 (65.484%)	
PA, n (%)			0.139
<600	2579 (72.322%)	212 (68.387%)	
>=600	987 (27.678%)	98 (31.613%)	
Ever taken birth control, n (%)			**<0.001**
No	872 (24.453%)	34 (10.968%)	
Yes	2694 (75.547%)	276 (89.032%)	
Regular period, n (%)			**<0.001**
No	1148 (32.193%)	195 (62.903%)	
Yes	2418 (67.807%)	115 (37.097%)	

Data are reported as the mean ± SD, Median (Q1-Q3) or n (%).

Mean ± SD or Median (Q1-Q3): P values were calculated by one-way ANOVA (normal distribution) and the Kruskal–Wallis H (skewed distribution) test, and the median (Q1-Q3) was used only when the mean value was less than double the standard difference.

PIR, poverty income ratio; BMI, body mass index; PA, physical activity.

Selenium Box-cox is in the quartile.

Q1 (-1.109 - 9.830), Q2 (9.831 - 11.375), Q3 (11.377 - 12.819), Q4 (12.820 - 29.253).

P-value less than 0.05 is expressed in bold.

### 3.2 Associations between dietary selenium and EMs

In order to further clarify the relationship between dietary selenium and EMs, this study was conducted using multivariate logistic regression equations, and the results, as shown in **Table 2**, showed a decreasing trend in the risk of developing EMs with increasing dietary selenium intake.

**Table 2:** Multivariate logistic regression.

Exposure	Non-adjusted		Adjust I		Adjust II	
	OR (95%CI)	P-value	OR (95%CI)	P-value	OR (95%CI)	P-value
Selenium Box-Cox	0.949 (0.907, 0.992)	0.020	0.944 (0.901, 0.989)	0.015	0.937 (0.887, 0.988)	0.016
Selenium Box-Cox group						
Q1	1.0[Ref]		1.0[Ref]		1.0[Ref]	
Q2	0.872 (0.640, 1.189)	0.386	0.878 (0.639, 1.205)	0.419	0.889 (0.640, 1.236)	0.485
Q3	0.680 (0.490, 0.945)	0.022	0.675 (0.483, 0.945)	0.022	0.694 (0.486, 0.991)	0.044
Q4	0.691 (0.498, 0.958)	0.027	0.668 (0.478, 0.934)	0.018	0.659 (0.449, 0.967)	0.033
Selenium Box-Cox group trend	0.871 (0.785, 0.967)	0.009	0.862 (0.774, 0.959)	0.006	0.859 (0.760, 0.971)	0.014

Table data: OR (95%CI) P value.

Outcome variable: Endometriosis.

Exposure variable: Selenium Box-Cox; Selenium Box-Cox group; Selenium Box-Cox group trend.

Non-adjusted model adjust for: None.

Adjust I model adjust for: Age; Race; PIR; Education level; Marital Status.

Adjust II model adjust for: Age; Race; PIR; Education level; Marital Status; BMI; Smoking Status; Alcohol Consumption; Hyperlipidemia; Hypertension; Theobromine; Vitamin B12; Carbohydrate; PA; Ever taken birth control; Regular period.

CI, confidence interval; OR, odds ratios; Ref, reference; PIR, poverty income ratio; BMI, body mass index; PA, physical activity.

Selenium Box-cox is in the quartile.

Q1 (-1.109 - 9.830), Q2 (9.831 - 11.375), Q3 (11.377 - 12.819), Q4 (12.820 - 29.253).

In this study, dietary selenium intake was divided into four categories: Q1 (-1.109 - 9.830), Q2 (9.831 - 11.375), Q3 (11.377 - 12.819) and Q4 (12.820 - 29.253). In the highest dietary selenium intake group (12.820 - 29.253), the unadjusted model showed a reduction in the risk of developing EMs of approximately 30.9% (OR = 0.691, 95% CI: 0.498, 0.958, p < 0.05), the adjusted Adjust 1 model showed a reduction in the risk of developing EMs of approximately 33.2% (OR = 0.668, 95% CI: 0.478, 0.934, p < 0.05), and the adjusted Adjust 2 model showed a reduction in the risk of developing EMs of about 34.1% (OR = 0.659, 95% CI: 0.449, 0.967, p < 0.05).

In addition, **Table 2** clearly illustrates the association between dietary selenium intake and EMs, with results showing a protective trend towards a lower risk of developing EMs as dietary selenium intake increases.

### 3.3 Smoothing curve fitting analysis and threshold effect analysis

**Figure 2:**
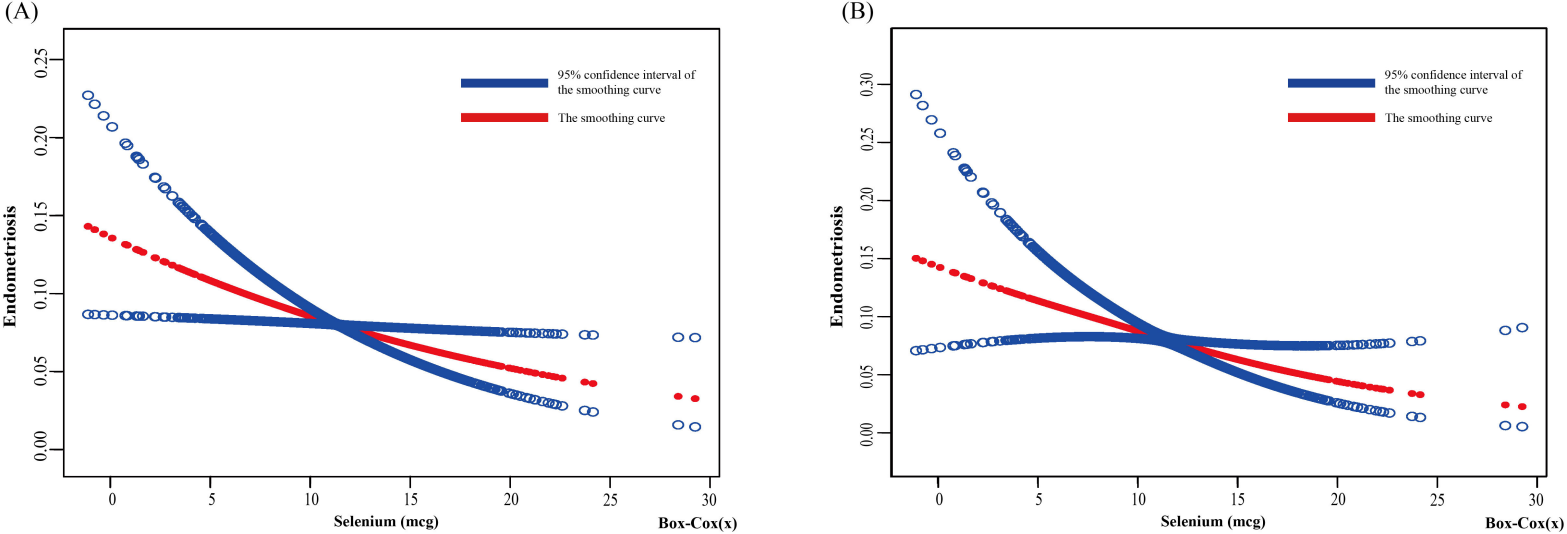
Relationship between dietary selenium and endometriosis risk by smooth curve fitting. The red line demonstrates the risk of endometriosis, and the blue ribbons illustrate its 95%CI. The X-axis is dietary selenium (continuous variable, Box-cox transformed), and the Y-axis is endometriosis. **(A)** Unadjusted; **(B)** Adjust for: Age; Race; PIR; Education level; Marital Status; BMI; Smoking Status; Alcohol Consumption; Hyperlipidemia; Hypertension; Theobromine; Vitamin B12; Carbohydrate; PA; Ever taken birth control; Regular period. CI, confidence interval; PIR, Poverty Income Ratio; BMI, body mass index; PA, Physical activity.

Based on the unadjusted and adjusted II models, this study used smooth curve fitting to depict the nonlinear relationship between dietary selenium intake and the risk of developing endometriosis (EMs), as illustrated in **Figure 2A** presents the smooth curve fitting based on the unadjusted model, while **Figure 2B** displays the smooth curve fitting based on the adjusted II model. Both figures reveal a negative correlation between dietary selenium intake and the likelihood of EMs, with higher dietary selenium intake being associated with a lower risk of EMs.

The results of the threshold effect analysis indicated a nonlinear relationship in the unadjusted model’s curve (log-likelihood ratio = 0.042), with an inflection point at 8.152 (**Table 3**). When the dietary selenium intake (after Box-Cox transformation) was less than 8.152, a significant negative correlation was observed between dietary selenium intake and EMs (OR: 0.917, 95% CI: 0.867, 0.971, p = 0.003). When the dietary selenium intake (after Box-Cox transformation) exceeded 8.152, no significant association was observed between dietary selenium intake and EMs (OR: 1.143, 95% CI: 0.928, 1.409, p = 0.210). In contrast, no nonlinear relationship was detected in the adjusted II model’s curve (log-likelihood ratio = 0.064).

**Table 3:** Threshold effect analysis of selenium with endometriosis.

Outcome: EMs	Unadjusted model	Adjusted model^a^
	OR (95%CI) P-value	OR (95%CI) P-value
Model I		
One line effect	0.949 (0.907, 0.992) 0.020	0.936 (0.887, 0.988) 0.016
Model II		
Turning point(K)	8.152	8.152
< K effect 1	1.143 (0.928, 1.409) 0.210	1.113 (0.900, 1.375) 0.324
> K effect 2	0.917 (0.867, 0.971) 0.003	0.905 (0.847, 0.967) 0.003
LRT test	0.042	0.064

Table data: OR (95%CI) P value.

Outcome variable: Endometriosis.

Exposure variable: Selenium Box-Cox.

EMs, endometriosis; OR, odds ratio; CI, confidence interval; LRT, log-likelihood ratio test; PA, physical activity.

a Adjusted for age, race, PIR, Education level, marital status, BMI, smoking status, alcohol consumption, hyperlipidemia, hypertension, theobromine, vitamin B12, carbohydrate, PA, ever taken birth control and regular period.

### 3.4 Subgroup analysis

**Figure 3:**
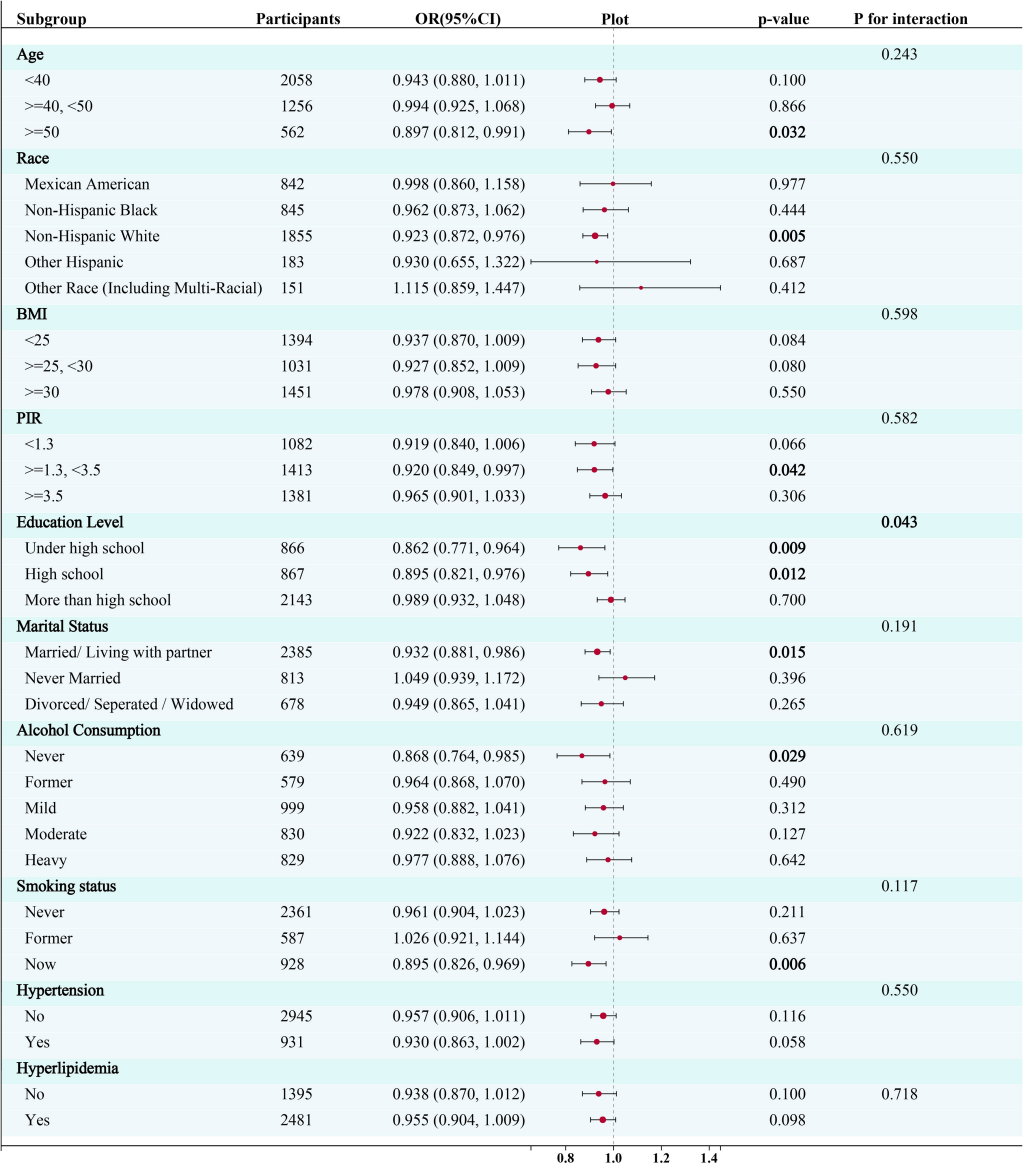
Subgroup analysis for the association between the Box-Cox transformed dietary selenium (continuous variable) and risk of endometriosis. CI, confidence interval; OR, odds ratios; PIR, Poverty Income Ratio; BMI, body mass index; PA, Physical activity.

To reduce heterogeneity, the present study further identified the relationship between EMs and a range of demographic and health-related variables and their interactions through stratified analyses. Stratified analyses were constructed based on age, race, PIR, BMI, education level, marital status, alcohol consumption, smoking status, hyperlipidemia and hypertension status. Refer to **Figure 3** for the results of the stratified analyses.

The logistic regression models revealed a significant negative relationship between selenium intake and the risk of endometriosis in participants aged fifty years and older (OR= 0.897, 95% CI: 0.812, 0.991; P=0.032), in non-Hispanic white participants (OR= 0.923, 95% CI: 0.872, 0.976; P=0.005), in participants with PIR >=1.3 and <3.5 (OR= 0.920, 95% CI: 0.849, 0.997; P=0.042), in participants with a high school education level (OR= 0.895, 95% CI: 0.821, 0.976; P=0.009) or under (OR= 0.862 95%CI:0.771, 0.964; P=0.012), in participants who get married or live with a partner (OR= 0.932, 95% CI: 0.881, 0.986; P=0.015), in participants who have never drunk (OR= 0.868, 95% CI: 0.764, 0.985; P=0.028), and in participants who smoke currently (OR= 0.895, 95% CI: 0.826, 0.969; P=0.006) (**Figure 3**). Furthermore, a significant interaction was observed between selenium intake and education level (p for interaction < 0.05), while no significant interaction was observed in any other subgroups.

**Figure 4:**
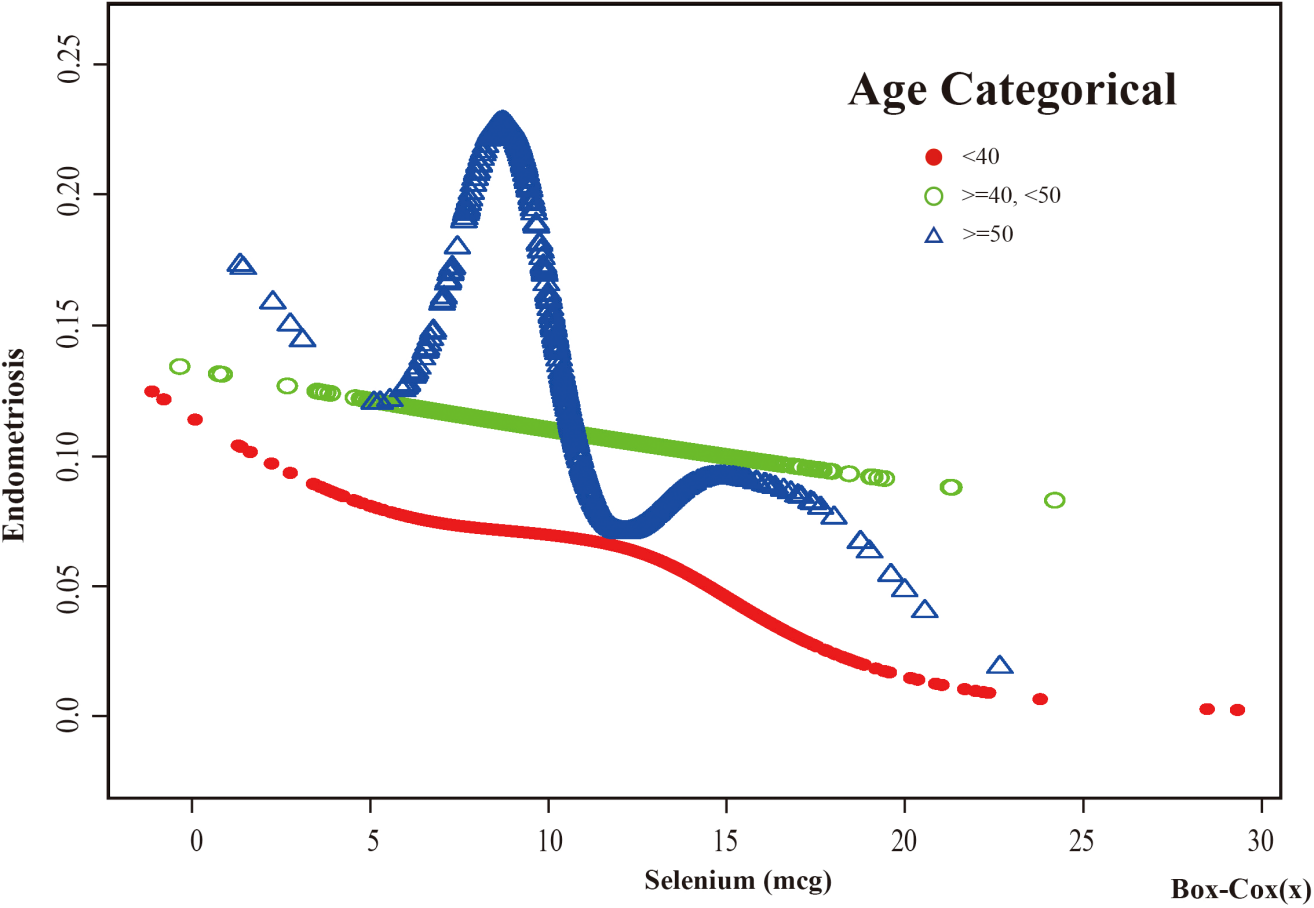
The association between Selenium and risk of EMs stratified by Age. In the age-stratified analysis, the red line represents age <40 years, the green line represents age >=40 or <50 years, and the blue line represents age >=50 years. Adjustment for race, PIR, BMI, education level, marital status, alcohol consumption, smoking status, hyperlipidemia, hypertension, theobromine intake, vitamin B12 intake, carbohydrate intake, PA, ever taken birth control and regular period. PIR, Poverty Income Ratio; BMI, body mass index; PA, Physical activity.

**Figure 5:**
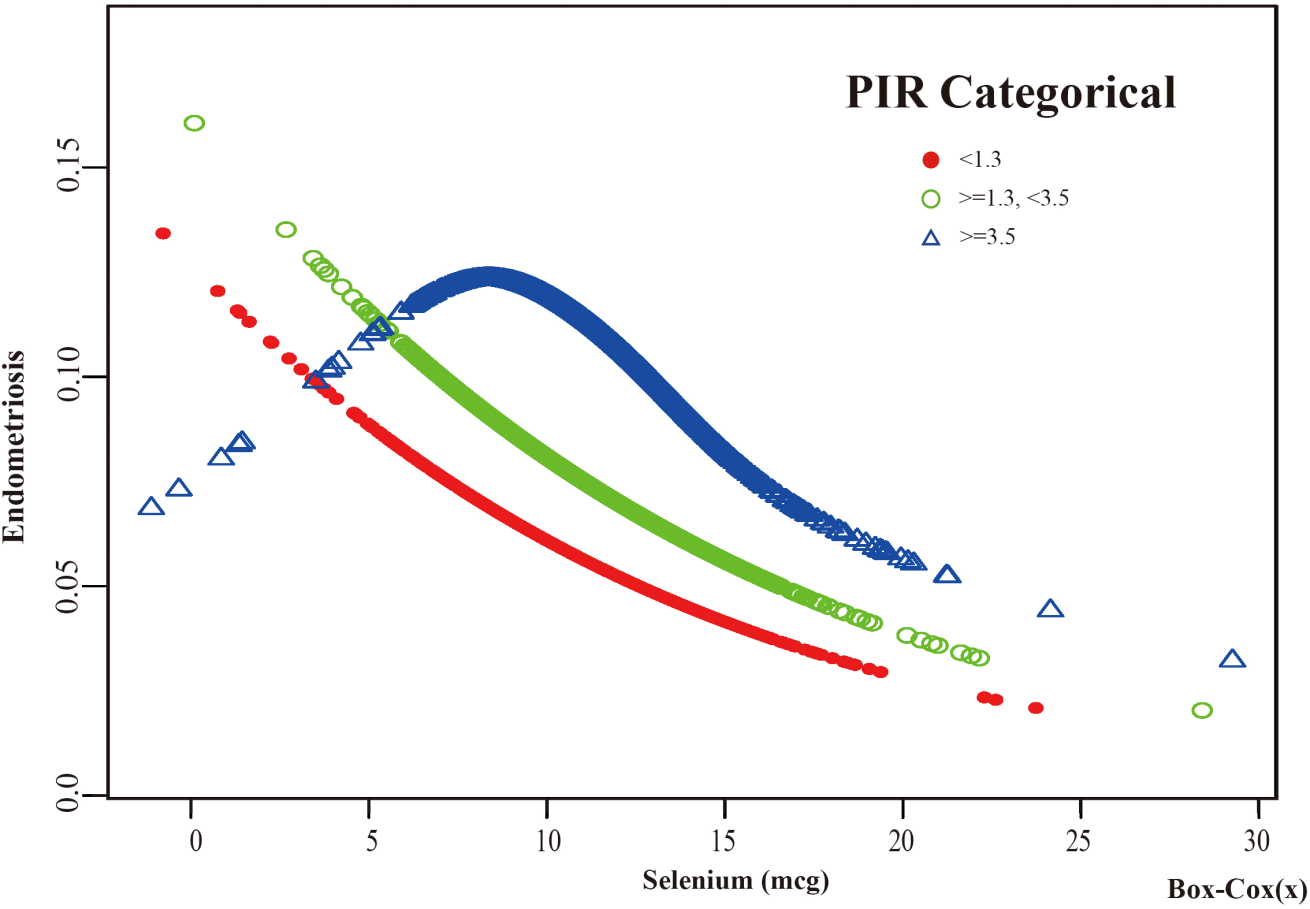
The association between Selenium and risk of EMs stratified by PIR. According to the PIR-stratified analysis, the red line represents a PIR <1.3, the green line represents a PIR >=1.3 or <3.5, and the blue line represents a PIR >=3.5. Adjustment for age, race, BMI, education level, marital status, alcohol consumption, smoking status, hyperlipidemia, hypertension, theobromine intake, vitamin B12 intake, carbohydrate intake, PA, ever taken birth control and regular period. PIR, Poverty Income Ratio; BMI, body mass index; PA, Physical activity.

**Figure 6:**
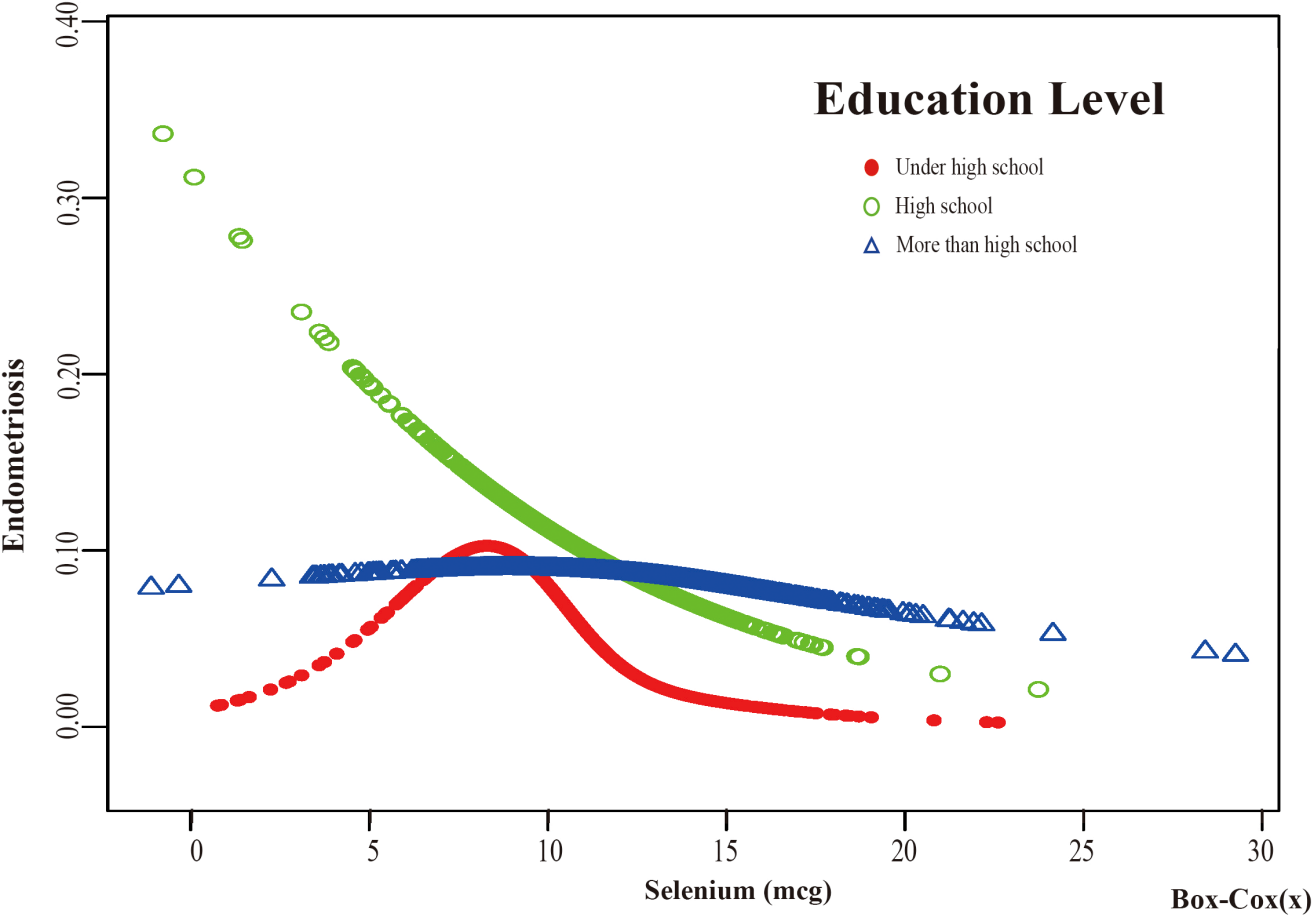
The association between Selenium and risk of EMs stratified by Education Level. According to the education level stratification analysis, the red line represents under high school, the green line represents high school, and the blue line represents more than high school. Adjustment for age, race, PIR, BMI, marital status, alcohol consumption, smoking status, hyperlipidemia, hypertension, theobromine intake, vitamin B12 intake, carbohydrate intake, PA, ever taken birth control and regular period. PIR, Poverty Income Ratio; BMI, body mass index; PA, Physical activity.

**Figure 7:**
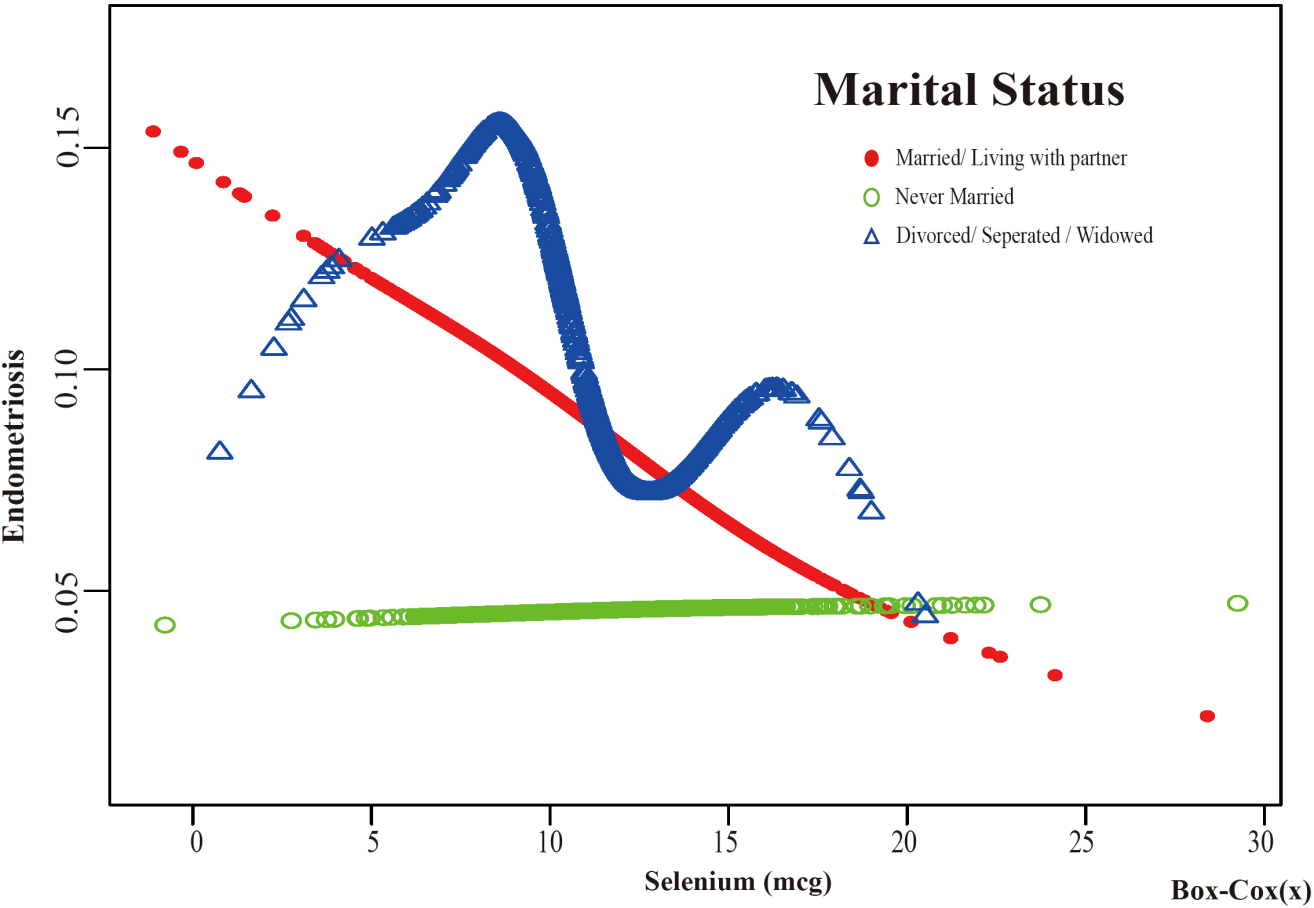
The association between Selenium and risk of EMs stratified by Marital Status. According to the education level stratification analysis, the red line represents Married/Living with a partner, the green line represents Never Married, and the blue line represents Divorced/Separated/Widowed. Adjustment for age, race, PIR, BMI, education level, alcohol consumption, smoking status, hyperlipidemia, hypertension, theobromine intake, vitamin B12 intake, carbohydrate intake, PA, ever taken birth control and regular period. PIR, Poverty Income Ratio; BMI, body mass index; PA, Physical activity.

**Figure 8:**
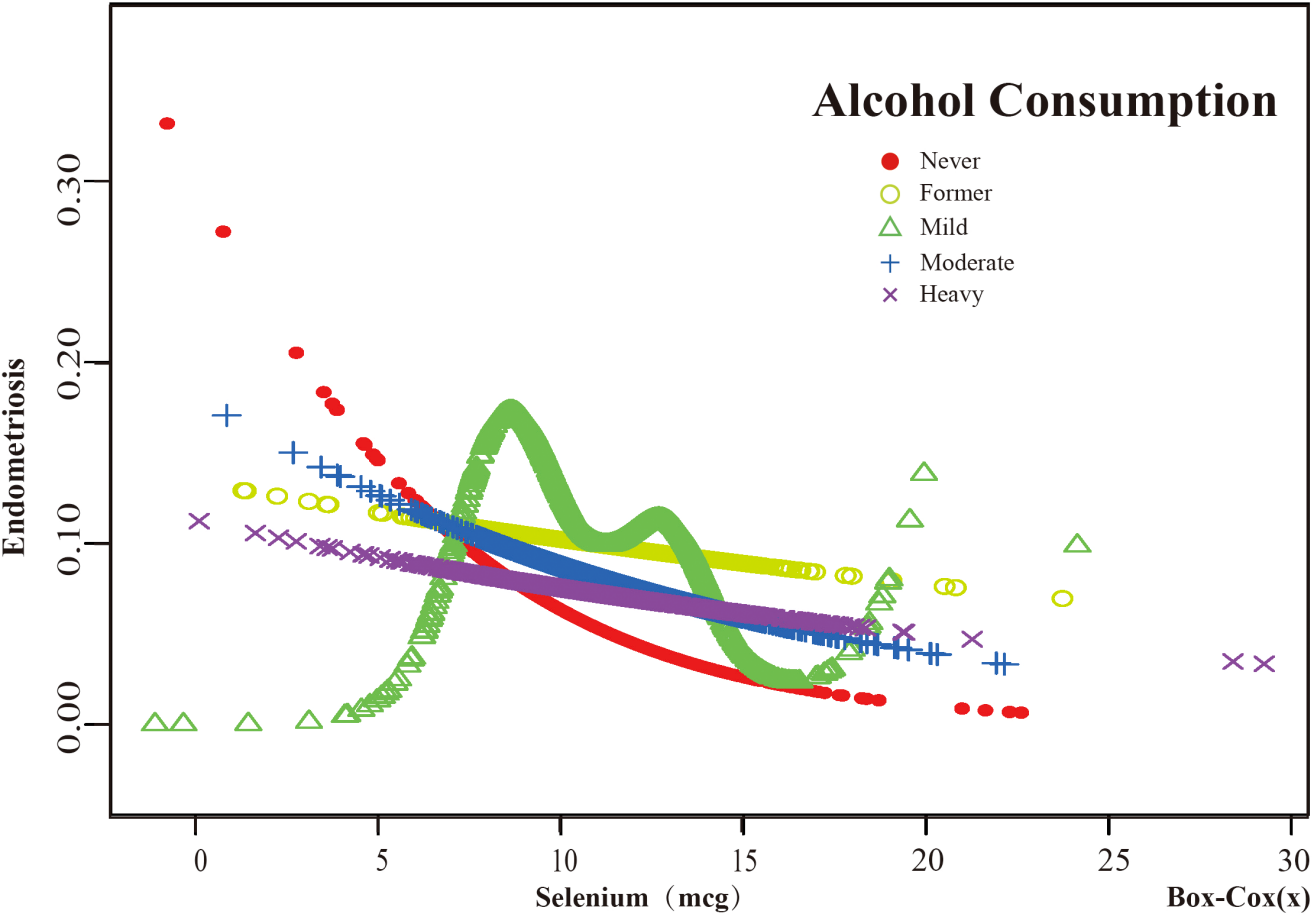
The association between Selenium and risk of EMs stratified by Alcohol Consumption. According to the results of the alcohol consumption stratification analysis, the red line represents Never, the yellow line represents Former, the green line represents Mild, the blue line represents Moderate, and the purple line represents Heavy. Adjustment for age, race, PIR, BMI, education level, marital status, smoking status, hyperlipidemia, hypertension, theobromine intake, vitamin B12 intake, carbohydrate intake, PA, ever taken birth control and regular period. PIR, Poverty Income Ratio; BMI, body mass index; PA, Physical activity.

**Figure 9:**
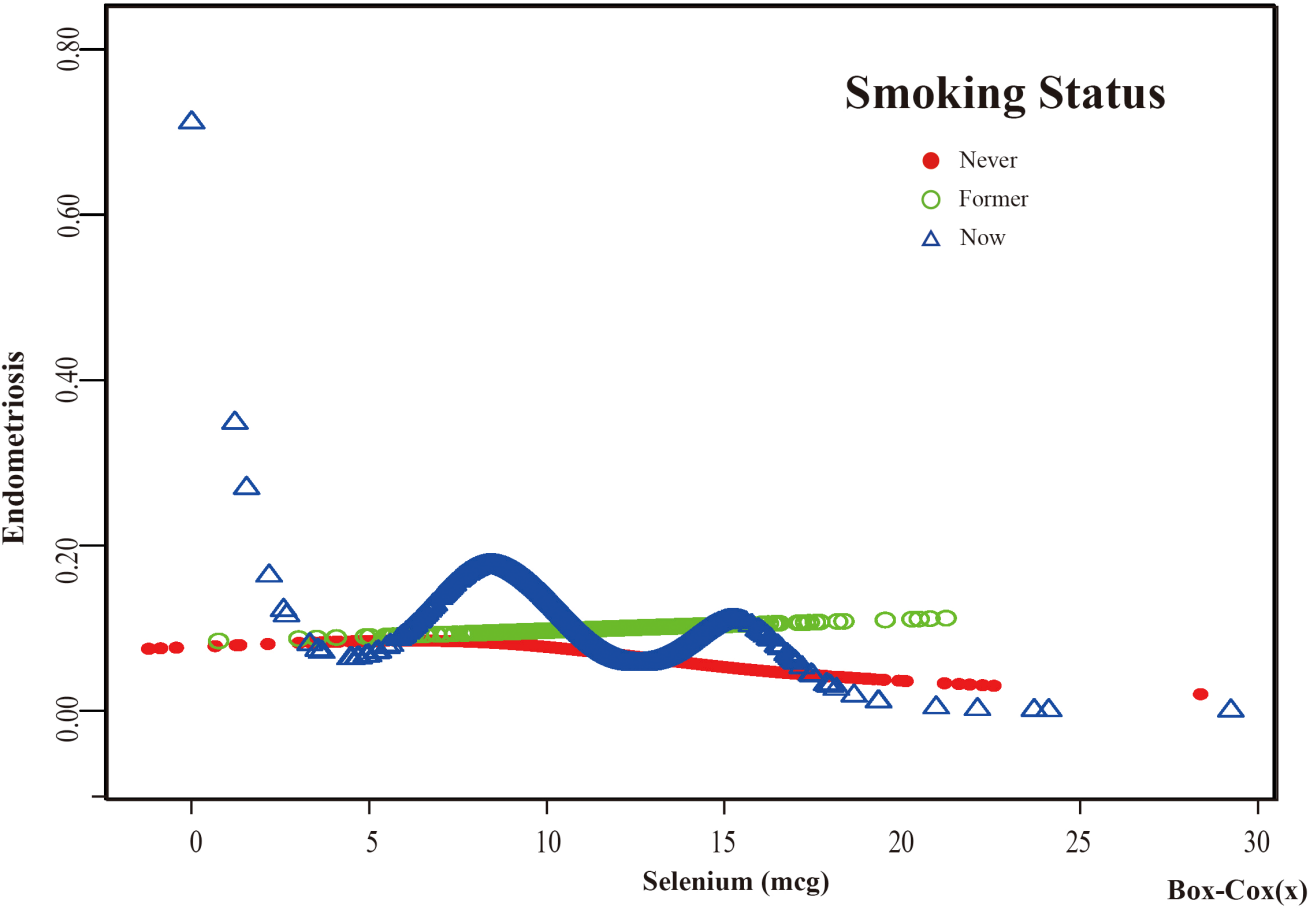
The association between Selenium and risk of EMs stratified by Smoking Status. In the smoking status stratification analysis, the red line represents never smoke, the green line represents former smoke, and the blue line represents now smoke. Adjustment for age, race, PIR, BMI, education level, marital status, alcohol consumption, hyperlipidemia, hypertension, theobromine intake, vitamin B12 intake, carbohydrate intake, PA, ever taken birth control and regular period. PIR, Poverty Income Ratio; BMI, body mass index; PA, Physical activity.

Based on these results, this study used smooth curve fitting to investigate the linear relationships between alcohol consumption, smoking status and the risk of EMs. Additionally, smooth curve fitting subgroup analyses were performed for the demographic variables of age, PIR, education level and marital status. Curve fitting results showed that as selenium intake levels increased, the risk of developing EMs tended to decrease for all participants except those aged 50 years and older, with a PIR greater than or equal to 3.5, with a high school education, less than a high school education, who were single women, non-drinkers, current smokers, and former smokers (**Figures 4**–**9**). Furthermore, trend analyses for other stratified groups reveal that with increasing selenium concentration, EMs initially show an increasing trend, followed by one or more turning points, displaying varying trends of increase and decrease.

Threshold effect analysis of stratified curves was performed, and the results are presented in **Supplementary Tables S1**, **S2** of **Supplementary Material S2**. A significant inflection point was observed in the “Under high school” subgroup of Education Level at 8.394 (log - likelihood ratio test=0.006). In this subgroup, when the dietary selenium intake (Box - cox transformed) was greater than 8.394, for each one - unit increase in dietary selenium intake (Box - cox transformed), the risk of endometriosis (EMs) was reduced by 39.5% (OR=0.605, 95%CI: 0.454, 0.805). Notably, the Education Level subgroup was the only one with significant interaction (p for interaction <0.05). In the “Mild” subgroup of Alcohol consumption, a significant inflection point was observed at 7.567 (log - likelihood ratio test=0.001). In this subgroup, when the dietary selenium intake (Box - cox transformed) was greater than 7.567, for each one - unit increase in dietary selenium intake (Box - cox transformed), the risk of EMs was reduced by 14.0% (OR=0.860, 95%CI: 0.756, 0.978). In the “ Never” subgroup of Smoking Status, a significant inflection point was observed at 7.633 (log - likelihood ratio test=0.006). In this subgroup, when the dietary selenium intake (Box - cox transformed) was greater than 7.633, for each one - unit increase in dietary selenium intake (Box - cox transformed), the risk of EMs was reduced by 10.4% (OR=0.896, 95%CI: 0.816, 0.983). In contrast, no significant relationships between dietary selenium intake and EMs were found in other subgroups before or after the potential inflection points.

### 3.5 Sensitivity analysis

In order to confirm the reliability of the findings, a sensitivity evaluation was executed in this investigation. Specifically, after eliminating extreme anomalies with dietary selenium intake surpassing 400, the multivariate logistic regression analysis was carried out again (**Table 4**). In the highest dietary selenium intake group, the unadjusted model showed a reduction in the risk of developing EMs of approximately 30.3% (OR = 0.697, 95% CI: 0.503, 0.966, p < 0.05), the adjusted Adjust 1 model showed a reduction in the risk of developing EMs of approximately 32.8% (OR = 0.672, 95% CI: 0.480, 0.940, p < 0.05), and the adjusted II model showed a reduction in the risk of developing EMs of about 33.8% (OR = 0.662, 95% CI: 0.451, 0.972, p < 0.05). In the sensitivity analysis, results similar to those of the original analysis were observed (**Table 2**), further corroborating the robustness of the results.

**Table 4:** Sensitivity analysis.

Exposure	Non-adjusted		Adjust I		Adjust II	
	OR (95%CI)	P-value	OR (95%CI)	P-value	OR (95%CI)	P-value
Selenium Box-Cox	0.951 (0.909, 0.994)	0.027	0.946 (0.902, 0.991)	0.019	0.938 (0.888, 0.991)	0.021
Selenium Box-Cox group						
Q1	1.0[Ref]		1.0[Ref]		1.0[Ref]	
Q2	0.872 (0.640, 1.189)	0.386	0.878 (0.639, 1.205)	0.419	0.888 (0.639, 1.235)	0.480
Q3	0.680 (0.490, 0.945)	0.021	0.675 (0.483, 0.945)	0.022	0.693 (0.485, 0.989)	0.044
Q4	0.697 (0.503, 0.966)	0.030	0.672 (0.480, 0.940)	0.020	0.662 (0.451, 0.972)	0.036
Selenium Box-Cox group trend	0.873 (0.786, 0.969)	0.010	0.863 (0.775, 0.961)	0.007	0.860 (0.761, 0.972)	0.016

Table data:OR (95%CI) P value.

Outcome variable: Endometriosis.

Exposure variable: Selenium Box-Cox; Selenium Box-Cox group; Selenium Box-Cox group trend.

Non-adjusted model adjust for: None.

Adjust I model adjust for: Age; Race; PIR; Education level; Marital Status.

Adjust II model adjust for: Age; Race; PIR; Education level; Marital Status; BMI; Smoking Status; Alcohol Consumption; Hyperlipidemia; Hypertension; Theobromine; Vitamin B12; Carbohydrate; PA; Ever taken birth control; Regular period.

CI, confidence interval; OR, odds ratio; Ref, reference; PIR, poverty income ratio; BMI, body mass index; PA, physical activity.

## 4 Discussion

The findings of this study revealed an inverse association between dietary selenium intake and the risk of EMs. Through multivariate logistic regression analysis, it was found that higher dietary selenium intake was linked to a decreased risk of EMs. Specifically, those in the group with the highest dietary selenium intake had a lower chance of having EMs.

EMs is a chronic, inflammatory gynecological disease. Prior studies have indicated that oxidative stress could be a pathogenic mechanism (2, 28). Current research indicates that GPX, a selenium-containing enzyme widely distributed in the uterine endometrial glandular epithelium, plays a crucial role in responding to oxidative stress and serves an important defensive role in addressing endometriosis (29). GPX is an important antioxidant enzyme that helps stop the accumulation of high concentrations of intracellular and extracellular peroxides (30). It can reduce potentially damaging ROS, such as hydrogen peroxide and lipid hydroperoxides, to harmless products like water and alcohols by coupling their reduction with the oxidation of glutathione (31). This process is crucial in maintaining cellular homeostasis and preventing oxidative damage (32). The effectiveness of GPX in this function is largely dependent on the availability of selenium, as it is a key component of the enzyme’s structure and activity (29, 33). Selenium, as a trace mineral and an essential nutrient in human diets (34), plays a vital role in the proper functioning of GPX. Dietary selenium plays an important role in inflammation and immunity, primarily through its incorporation into selenoproteins (35). The relationship between seleniumproteins and health has garnered attention in recent decades due to the identification of polymorphisms in seleniumprotein genes linked to disease (36). Yavuz et al. found a negative correlation between EMs and glutathione peroxidase (37), which significantly increases with higher dietary selenium intake (38–42). Furthermore, certain plant-derived bioactive substances like resveratrol have received the majority of attention in current research on plant-derived medicines for the treatment of EMs. The mechanisms by which these agents exert their influence encompass a multifaceted impact on established signaling mediators, such as matrix metalloproteinases, ROS, and proteins implicated in apoptosis (43). This therapeutic approach targets endometriosis by modulating pathways associated with oxidative stress and lipid peroxidation (44) (45). In summary, the significant role of glutathione peroxidase, a selenium-containing enzyme, in the research on the pathogenesis of EMs suggests a significant link between dietary selenium and EMs.

However, it is important to note that some studies have suggested that excess selenium intake may be harmful. For example, a study by Ma XM et al. found a significant positive association between dietary selenium intake and the incidence of type 2 diabetes (T2DM) in a fully adjusted model (OR = 1.49, 95% CI: 1.16, 1.90, p = 0.0017) (46). Another study by Pi Y et al. found that higher dietary selenium intake was associated with a lower risk of chronic kidney disease (CKD), but the relationship was not linear and may involve a threshold effect (47). These findings indicate that the effects of dietary selenium intake are not solely positive and may depend on the intake level. The study indicated a nonlinear relationship in the unadjusted model’s curve, with an inflection point at 8.152. When the dietary selenium intake (after Box-Cox transformation) was less than 8.152, a significant negative correlation was observed between dietary selenium intake and EMs. These findings suggest that within a certain range, higher dietary selenium intake is associated with a reduced risk of EMs. This indicates that the protective effects of selenium may be observed at moderate levels of intake, and further research is needed to determine the optimal range for selenium intake in the context of EMs risk.

Subgroup analysis identified certain demographic groups where the association between dietary selenium intake and EMs risk was more pronounced. These groups included participants aged 50 years and older, non-Hispanic white participants, participants with a PIR between 1.3 and 3.5, participants with a high school education or less, married or cohabiting participants, participants who had never consumed alcohol, and current smokers. Additionally, a significant interaction was observed between selenium intake and education level, suggesting that the relationship between selenium intake and EMs risk may vary across different education levels.

Previous studies have indicated that age is a significant factor in the development of EMs. For instance, Broi et al. have mentioned that EMs is a very common disease among women of reproductive age (48). Yasui et al. pointed out that women with a history of infertility, especially those with EMs, have a significantly earlier menopause age compared to women without such a history (49). Hemmert et al. found that women with endometriosis are more likely to be younger (50). These studies indicate a significant direct association between a woman’s age and EMs. The protective effect of selenium in older women may be due to age-related changes in hormonal levels and reproductive health. Additionally, race has been identified as a risk factor for EMs. In general, White patients have a greater probability of receiving an EMs diagnosis than non-White patients (51). Patients of other racial backgrounds faced higher mortality rates and healthcare costs in contrast to White patients, who benefited from better medical resources (52). The protective effect of selenium in non-Hispanic white participants may be related to genetic factors, lifestyle differences, or access to healthcare.

The protective effect of selenium in participants with 1.3≤ PIR< 3.5 may be related to differences in lifestyle, diet, and access to healthcare. Higher socioeconomic class and higher occupational levels have been linked to an increased incidence of endometriosis in women, according to numerous early epidemiologic studies on endometriosis risk factors (53, 54). Nevertheless, Hu et al. research uncovers heightened risk of endometriosis in populations with lower incomes (18). Moreover, some studies have indicated that married women may experience distinct psychosocial pressures due to reproductive stress or family responsibilities, which may potentially impact their health status to some degree. For instance, one study posits that married women may be more vulnerable to fertility-related expectations and social pressures compared to single women (55). The connection between alcohol and EMs remains controversial; some studies have shown a significant correlation between alcohol consumption and the occurrence of EMs, possibly due to alcohol’s effect on estrogen levels and immune function in the body (56), but other studies have not found a significant association (54, 57). The research revealed an inverse relationship between never drinking and EMs. The differences in study results could be due to alcohol’s dual role in EMs, as a pain and stress reliever for patients (where it is used as a form of self-management for pain and stressful events, or as a manifestation of psychiatric comorbidities) while exacerbating inflammation and oxidative stress through habitual heavy drinking (58). Additionally, smoking not only disrupts immune-inflammatory balance by modulating cytokines like TNF-α and IL-6 but also exacerbates conditions such as EMs by triggering an overexpression of pro-inflammatory genes, which can lead to the dysregulation of selenium-mediated anti-inflammatory responses and contribute to the pathogenesis of the disease (10, 56, 59).

The interaction test in the subgroup analysis showed that only education level significantly interacted with selenium and EMs (p for interaction < 0.05). Specifically, in women with a high school education or less, a negative association between dietary selenium intake and EMs was observed. This suggests that the protective effect of selenium may vary across different education levels. One study observed that patients’ health literacy, encompassing the capacity to identify, comprehend, assess, and utilize health information, is pivotal to the management of chronic illnesses such as endometriosis (60).

This study indicates that higher dietary selenium intake may reduce EMs risk in certain groups, but the results are limited by the cross-sectional NHANES design, which cannot prove causality. Other limitations include the inability to control for all confounders, the exclusion of participants under 20, undiagnosed EMs cases in controls due to self-reported data, and the study’s limited applicability to American citizens due to NHANES sample selection. This necessitates further research on racial and geographic disparities. The study was conducted using a questionnaire-based self-reported dietary recall, and some errors may occur in the self-reported dietary intake. Given the significant day-to-day variations in dietary intake, dietary recall bias may exist in the measurement of individual dietary components. Relying on self-reported data for the diagnosis of endometriosis has limitations, and the potential for misclassification bias is recognized, especially considering that many cases may remain undiagnosed, particularly among individuals with asymptomatic or mild symptoms. The NHANES database source has limitations, leading to the omission of key covariates such as family history of endometriosis and history of pelvic surgery. The study also did not account for overall diet quality, which could influence the observed association between selenium intake and EMs risk. The lack of significant links in younger age groups, other races, and higher education subgroups may be due to factors like selenium metabolism differences, healthcare access, lifestyle, and small sample sizes. No clear association was found between EMs and hypertension/hyperlipidemia, possibly due to shared risk factors, complex EMs mechanisms, and study limitations. While some studies have reported a negative correlation between BMI and EMs risk, this study did not find a significant association, which may be due to differences in the sample population and the limitations of BMI as a body fat indicator. Further research is needed to explore these.

Furthermore, this study also has certain strengths. First, it found a negative correlation between selenium and EMs, indicating that balanced dietary selenium intake helps reduce the risk of EMs. Providing scientific dietary guidance for EMs patients is important. Second, many potential confounders and influencing factors were adjusted in this study, enhancing the accuracy of the results and promoting precise analysis of the study objective. This also deepened the current understanding of the relationship between selenium and EMs. Third, compared to previous studies, this study had a larger sample size, utilizing data from NHANES from 1999 to 2006, including 39,352 potential participants, of which 3876 were included in this study. Additionally, a sensitivity analysis was conducted, which further confirmed the robustness of the results.

## 5 Conclusions

Our findings suggest a negative correlation between dietary selenium intake and endometriosis risk. However, potential confounding factors may influence this association. Given the limitations of this cross-sectional study, such as reliance on self-reported data, further prospective research is required to confirm causality and explore underlying mechanisms.

## Data Availability

Publicly available datasets were analyzed in this study. This data can be found here: https://www.cdc.gov/nchs/nhanes.
