# Supplementary material for: Association between dietary selenium intake and endometriosis risk: a cross-sectional analysis
Source: Front Endocrinol (Lausanne). 2025 Jun 30;16:1486790. doi: 10.3389/fendo.2025.1486790 (PMC12256242; doi:10.3389/fendo.2025.1486790)
Supplement: Supplementary file 1 [file DataSheet1.docx]

**Supplementary Material S1**
The Box-Cox transformation (transforming the value x to the new value y, given a parameter commonly designated by λ) is defined as follows (1,2):


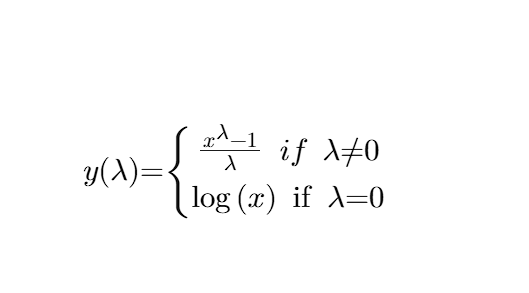


The transformation acts differently depending on the value of λ. For certain values of λ, the transformation is equivalent to other well-known transformations **(Figure S1)**; for instance, if λ = 0, it turns to a log-transformation. But, which value of λ is better and makes the distribution of the transformed data closer to a normal distribution? To determine the most appropriate value for λ, we may use the function boxcox from the R package EnvStats (3). The most appropriate value for λ for our data set was 0.369. The transformation is then:

(X^0.369 -1)/0.369


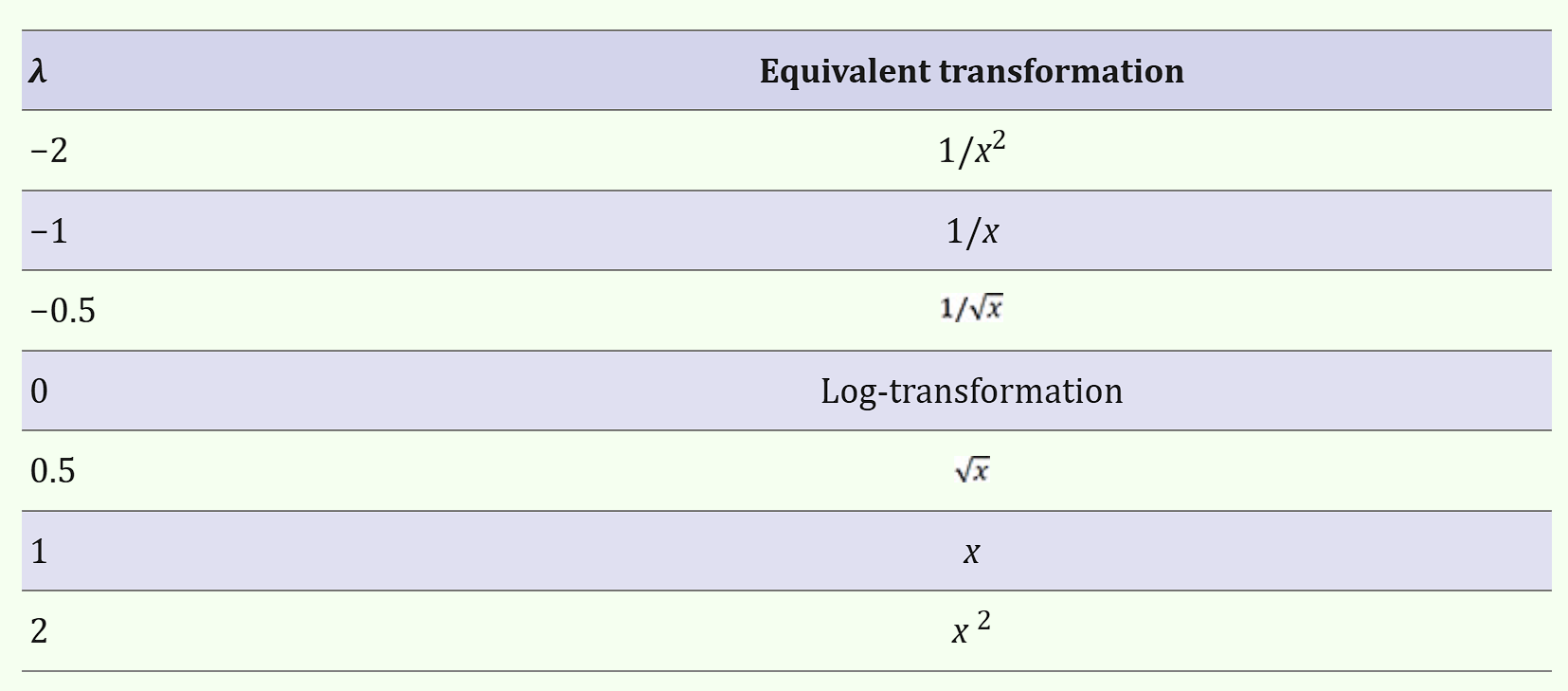


**Figure S1.** Equivalent transformations for certain values of λ in a Box-Cox transformation.

As own in the histogram of transformed dietary selenium intake **(Figure S2)**, the transformed data distribution shows approximately normal distribution.


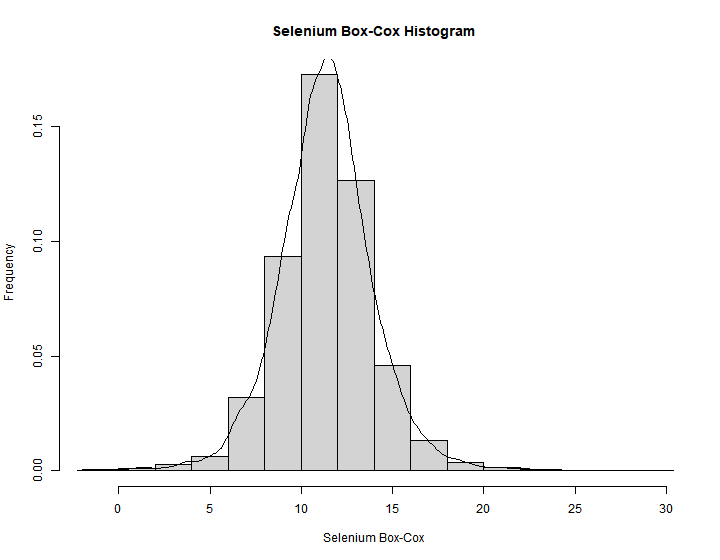


**Figure S2.** The histogram of Box-cox transformed dietary selenium intake.

# References

1. Box GEP, Cox DR. An analysis of transformations. J R Stat Soc Ser B-Methodol. 1964;26(2):211–43.

2. Habibzadeh F. Data distribution: normal or abnormal? J Korean Med Sci. 2024 Jan 15;39(3):e35.

3. Millard SP. EnvStats: an R package for environmental statistics [Internet]. New York, NY: Springer; 2013 [cited 2025 Feb 8]. Available from: https://link.springer.com/10.1007/978-1-4614-8456-1
