# Supplementary material for: Association between dietary selenium intake and endometriosis risk: a cross-sectional analysis
Source: Front Endocrinol (Lausanne). 2025 Jun 30;16:1486790. doi: 10.3389/fendo.2025.1486790 (PMC12256242; doi:10.3389/fendo.2025.1486790)
Supplement: Supplementary file 2 [file DataSheet2.docx]

**Table S1.** Threshold effect analysis of selenium with endometriosis, stratified by age, PIR, education level, and marital status.

| Outcome: EMs | Age | | | PIR | | | Education Level | | | Marital Status | | |
| --- | --- | --- | --- | --- | --- | --- | --- | --- | --- | --- | --- | --- |
|  | <40 | >=40, <50 | >=50 | <1.3 | >=1.3, <3.5 | >=3.5 | Under high school | High school | More than high school | Married/ Living with partner | Never Married | Divorced/ Seperated / Widowed |
|  | OR (95%CI) P-value | OR (95%CI) P-value | OR (95%CI) P-value | OR (95%CI) P-value | OR (95%CI) P-value | OR (95%CI) P-value | OR (95%CI) P-value | OR (95%CI) P-value | OR (95%CI) P-value | OR (95%CI) P-value | OR (95%CI) P-value | OR (95%CI) P-value |
| Model I |  |  |  |  |  |  |  |  |  |  |  |  |
| One line effect | 0.923 (0.843, 1.010) 0.0807 | 1.004 (0.920, 1.095) 0.9324 | 0.959 (0.830, 1.107) 0.5656 | 0.940 (0.823, 1.073) 0.3568 | 0.895 (0.807, 0.992) 0.0349 | 0.965 (0.891, 1.045) 0.3796 | 0.781 (0.654, 0.933) 0.0064 | 0.902 (0.796, 1.023) 0.1085 | 0.985 (0.920, 1.054) 0.6555 | 0.954 (0.888, 1.025) 0.1991 | 0.952 (0.814, 1.112) 0.5312 | 0.921 (0.813, 1.043) 0.1965 |
| Model II |  |  |  |  |  |  |  |  |  |  |  |  |
| Turning point(K) | 14.182 | 7.592 | 8.096 | 15.424 | 9.701 | 7.697 | 8.394 | 6.969 | 8.152 | 8.221 | 14.043 | 12.635 |
| < K effect 1 | 0.971 (0.873, 1.080) 0.5916 | 1.632 (0.857, 3.109) 0.1362 | 1.302 (0.788, 2.152) 0.3037 | 0.967 (0.842, 1.110) 0.6319 | 0.942 (0.751, 1.180) 0.6021 | 2.666 (0.836, 8.503) 0.0976 | 1.497 (0.755, 2.970) 0.2481 | 0.743 (0.509, 1.086) 0.1256 | 1.355 (0.882, 2.081) 0.1651 | 1.140 (0.882, 1.475) 0.3166 | 1.154 (0.908, 1.467) 0.2405 | 0.885 (0.764, 1.024) 0.1011 |
| > K effect 2 | 0.481 (0.226, 1.025) 0.0578 | 0.960 (0.868, 1.063) 0.4341 | 0.901 (0.757, 1.071) 0.2366 | 0.000 (0.000, Inf) 0.8989 | 0.872 (0.756, 1.007) 0.0616 | 0.912 (0.829, 1.002) 0.0563 | 0.605 (0.454, 0.805) **0.0006** | 0.936 (0.812, 1.080) 0.3638 | 0.952 (0.878, 1.031) 0.2276 | 0.918 (0.840, 1.002) 0.0566 | 0.439 (0.174, 1.107) 0.0811 | 1.052 (0.788, 1.406) 0.7296 |
| LRT test | **0.019** | 0.055 | 0.149 | **0.039** | 0.607 | **0.007** | **0.006** | 0.317 | 0.074 | 0.108 | **0.015** | 0.342 |
| Table data: OR (95%CI) P value  Outcome variable: Endometriosis  Exposure variable: Selenium Box-Cox Abbreviations: EMs, endometriosis; OR, odds ratio; CI, confidence interval; LRT, log-likelihood ratio test; PA: physical activity Adjusted for age, race, PIR, Education level, marital status, BMI, smoking status, alcohol consumption, hyperlipidemia, hypertension, theobromine, vitamin B12, carbohydrate, PA, ever taken birth control and regular period except the stratification. | | | | | | | | | | | | |

**Table S2.** Threshold effect analysis of selenium with endometriosis, stratified by alcohol consumption and smkoing status.

| Outcome: EMs | Alcohol consumption | | | | | Smoking Status | | |
| --- | --- | --- | --- | --- | --- | --- | --- | --- |
|  | Never | Former | Mild | Moderate | Heavy | Never | Former | Now |
|  | OR (95%CI) P-value | OR (95%CI) P-value | OR (95%CI) P-value | OR (95%CI) P-value | OR (95%CI) P-value | OR (95%CI) P-value | OR (95%CI) P-value | OR (95%CI) P-value |
| Model I |  |  |  |  |  |  |  |  |
| One line effect | 0.793 (0.644, 0.976) 0.0289 | 1.047 (0.909, 1.206) 0.5244 | 0.949 (0.857, 1.051) 0.3169 | 0.953 (0.832, 1.092) 0.4880 | 0.926 (0.821, 1.044) 0.2094 | 0.940 (0.868, 1.017) 0.1237 | 0.984 (0.858, 1.129) 0.8181 | 0.901 (0.807, 1.006) 0.0633 |
| Model II |  |  |  |  |  |  |  |  |
| Turning point(K) | 6.708 | 12.826 | 7.567 | 13.739 | 15.75 | 7.633 | 13.269 | 8.536 |
| < K effect 1 | 0.605 (0.334, 1.093) 0.0960 | 1.013 (0.848, 1.210) 0.8861 | 5.733 (0.904, 36.361) 0.0639 | 0.997 (0.849, 1.171) 0.9709 | 0.944 (0.833, 1.071) 0.3734 | 1.489 (0.836, 2.653) 0.1765 | 1.072 (0.892, 1.288) 0.4593 | 1.056 (0.774, 1.440) 0.7329 |
| > K effect 2 | 0.827 (0.660, 1.036) 0.0982 | 1.144 (0.828, 1.581) 0.4150 | 0.860 (0.756, 0.978) **0.0215** | 0.728 (0.432, 1.227) 0.2332 | 0.488 (0.092, 2.591) 0.3997 | 0.896 (0.816, 0.983) **0.0206** | 0.756 (0.501, 1.140) 0.1824 | 0.856 (0.740, 0.989) 0.0355 |
| LRT test | 0.399 | 0.563 | **0.001** | 0.229 | 0.261 | **0.029** | 0.125 | 0.246 |
| Table data: OR (95%CI) P value  Outcome variable: Endometriosis  Exposure variable: Selenium Box-Cox Abbreviations: EMs, endometriosis; OR, odds ratio; CI, confidence interval; LRT, log-likelihood ratio test; PA: physical activity Adjusted for age, race, PIR, Education level, marital status, BMI, smoking status, alcohol consumption, hyperlipidemia, hypertension, theobromine, vitamin B12, carbohydrate, PA, ever taken birth control and regular period except the stratification. | | | | | | | | |
